# The impact of physical activity on self-emotion management among university students in Western China: the mediating roles of self-rated health and life satisfaction

**DOI:** 10.3389/fpsyg.2025.1567576

**Published:** 2025-04-29

**Authors:** Xin-yu Zhang, Yang-Sheng Zhang, Shan-shan Han, Garry Kuan, Hu Lou, Fan-zheng Mu, Wei-dong Zhu, Yu-peng Ye, Ya-xing Li, Shu-qiao Meng, Shuo Feng, Han Li, Zhong-lei Cui, Yao Zhang, Qing Zhang, Guang-xu Wang, Lin-lin Zhao, Bao-wei Zhou, Yong Wei, Bo Li

**Affiliations:** ^1^Institute of Sports Science, Nantong University, Nantong, China; ^2^School of Physical Education, Nanjing Xiaozhuang University, Nanjing, Jiangsu, China; ^3^Athletic Training Academy, Chengdu Sport University, Chengdu, China; ^4^Exercise and Sports Science Programme, School of Health Sciences, Universiti Sains Malaysia, George Town, Malaysia; ^5^School of Physical Education, Jinggangshan University, Ji'an, China; ^6^Physical Education College, Shangqiu University, Shangqiu, China; ^7^Department of Physical Education, Xidian University, Xi'an, Shaanxi, China; ^8^College of Physical Education, Xinyang Normal University, Xinyang, Henan, China; ^9^Department of Physical Education, Ordos Institute of Applied Technology, Ordos, China; ^10^Physical Education College of Shangqiu Normal University, Shangqiu, China; ^11^Institute of Sports and Health, Zhengzhou Shengda University, Zhengzhou, China; ^12^Department of Physical Education, Yangling Vocational and Technical College, Yangling, Shaanxi, China; ^13^College of Physical Education, Henan Normal University, Xinxiang, China; ^14^School of Physical Education, Shanghai Normal University, Shanghai, China; ^15^School of Physical Education and Health, Changzhou Liu Guojun Vocational Technology College, Changzhou, China; ^16^Nantong Institute of Rehabilitation Medicine, Nantong Rehabilitation Hospital, Nantong, Jiangsu, China; ^17^Nantong Institute of Technology, Nantong, Jiangsu, China

**Keywords:** physical activity, self-emotion management, self-rated health, life satisfaction, mediating effect

## Abstract

**Objective:**

This study aims to investigate the impact of Physical Activity on Self-Emotional Management among university students and to verify the mediating roles of Self-Rated Health and Life Satisfaction.

**Methods:**

A stratified cluster sampling method was used to enroll 10,300 university students from Western China. Questionnaires were used to collect data, which included demographic and sociological information, the Physical Activity Scale (for measuring Physical Activity), the Emotional Intelligence Scale (for measuring Self-Emotional Management), the Short-Form Health Survey (for measuring Self-Rated Health), and the Satisfaction with Life Scale (for measuring Life Satisfaction). The results were statistically analyzed by using SPSS 26.0.

**Results:**

There was a positive correlation between different intensities of Physical Activity and Self-Emotional Management among university students in Western China (*p* < 0.001). Yet the direct predictive effect on Self-Emotional Management was not significant. Low-Intensity Physical Activity positively predicted Self-Rated Health (*β* = 0.876, *p* < 0.001), while Vigorous-Intensity Physical Activity negatively predicted Self-Emotional Management (*β* = −1.500, *p* < 0.001). Self-Rated Health positively predicted both Life Satisfaction and Self-Emotional Management (*β* = 0.118, *p* < 0.001; *β* = 0.030, *p* < 0.001), and Life Satisfaction positively predicted Self-Emotional Management (*β* = 0.403, *p* < 0.001). Self-Rated Health partially mediated the relationship between Low-Intensity Physical Activity/Vigorous-Intensity Physical Activity and Self-Emotional Management, with effect sizes of 0.026 (95% CI: 0.002–0.052) and −0.045 (95% CI: −0.080 to −0.010) respectively. The serial mediation pathway, the Low-Intensity Physical Activity/Vigorous-Intensity Physical Activity→Self-Rated Health→Life Satisfaction→Self-Emotional Management was significant (*p* < 0.05).

**Conclusion:**

Low-Intensity Physical Activity or Vigorous-Intensity Physical Activity cannot directly influence university students’ Self-Emotional Management ability. Based on self-determination theory, Self-Rated Health can independently influence university students’ Self-Emotional Management ability. It can also indirectly influence Self-Emotional Management by incorporating the chained mediation effect of Self-Rated Health and Life Satisfaction.

## 1 Introduction

Emotion lies at the core of human experience. It represents the physiological and psychological responses to external environments and internal states (Hajiyeva and Kosonogov, 2023). Self-Emotional Management (SEM) denotes the scientific intervention in recognizing, expressing, experiencing, and regulating one’s emotions to promote physical and mental health (Morales et al., 2023). University years are crucial for character formation. Enhancing students’ SEM abilities is vital for cultivating good character during this period. Mental health does not merely refer to an individual’s absence of symptoms or diseases. Instead, it signifies that an individual enjoys a sound physical and mental state and a psychological state that is well-adapted to society (World Health Organization, 1950). As society develops rapidly, academic and employment competition among university students has become more intense. This has substantially increased their stress levels, giving rise to negative emotions. Students with poor SEM skills are more prone to psychological problems. In severe cases, these problems can even be life-threatening (Hu et al., 2016).

The “2022 Survey Report on the Mental Health of Chinese University Students” reveals that 21.48% of Chinese university students are at risk of different degrees of depression, and 45.28% may face the risk of anxiety (Fu and Zhang, 2022) WHO statistics show that the prevalence of mental health problems in China is increasing (Chinese Government Website, 2023). A recent study in The Lancet found that about 173 million people in China are afflicted with mental disorders, such as anxiety, depression, and obsessive-compulsive disorder. Around 158 million have never received professional treatment, and only 15 million, <10%, have sought treatment. Only 15 million, <10%, have sought treatment (Lu et al., 2021). In recent years, there has been an increasing emphasis on the mental health of university students. The “Special Action Plan for Strengthening and Improving Students’ Mental Health Work in the New Era” has been introduced (World Health Organization, 2022). This policy aims to boost students’ mental health, enhance their emotional management skills, and promote their all-round development. Currently, SEM is a key research area for scholars globally. They mainly explore the relationship between emotions and behavior.

Physical Activity (PA) involves bodily movements that increase energy expenditure above resting metabolic levels (Anshel et al., 1991). Regular participation in PA offers several benefits for university students. It enhances physical fitness and boosts immunity. Regular involvement in PA offers several benefits for university students, including enhanced physical fitness, improved cardiovascular health, and reduced cognitive decline from poor sleep (Haddad et al., 2024). Research suggests that PA is linked to improvements in at least one of the four dimensions of university students’ quality of life: physical health, mental health, environmental vitality, and social interaction (Sette Abrantes et al., 2022). The mental health benefits of engaging in PA have drawn substantial attention and research from both domestic and international scholars. Recent studies indicate that PA intervention not only improves mood through physiological mechanisms but also indirectly alleviates psychological distress by enhancing self-rated mental health and optimizing dietary patterns, particularly evident among women and younger adults (Levante et al., 2024). Research has demonstrated that positive emotions are crucial protective factors for mental health (Tugade et al., 2004). Based on the cognitive-behavioral hypothesis, PA can enhance psychological and social mechanisms such as self-esteem and social support (Deng et al., 2023), improve emotional states, and induce positive emotions. Strengthening emotional regulation abilities and a sense of social belonging alleviates negative emotions like depression and anxiety, thereby protecting the mental health of college students. This effect is particularly prominent in group exercise settings (White et al., 2024). Emotional health is one indicator of mental wellbeing. Compared to college students who do not actively participate in PA, those who actively engage in PA effectively convert positive experiences during PA into long-term psychological benefits through emotional regulation. These students exhibit significantly lower levels of depression and higher levels of mental health (Wenig et al., 2023). Thus, there is a close relationship between PA and emotions.

Extensive international studies have demonstrated that the multifaceted regulatory mechanisms of PA on individual emotions, under the influence of various factors such as enhancing executive function, strengthening psychological resilience, improving life satisfaction, and fulfilling basic psychological needs-oriented towards health, emphasize the positive impact of PA on emotional wellbeing (Chen et al., 2024; Díaz et al., 2024; Savikangas et al., 2024). A significant imbalance becomes evident when focusing on regional differences in China. The Western region of China has distinct characteristics in terms of economic development and ethnic distribution, which exhibit significant disparities and offer special research value. University students’ participation in physical activities shows a gradient in the eastern, central, and western regions. The eastern region has the highest participation rate at 39.2%, followed by the central region at 30.1%, and the west region at the lowest at 25.8% (Cai et al., 2009). This trend aligns with China’s current regional socio-economic development levels. There is a greater focus on emotion-related studies in the eastern and central regions (Meng et al., 2015; Wei et al., 2022; Liu W. J. et al., 2023). The fast pace of development, intense employment competition, and high academic and life pressures in these regions lead to significant emotional issues. Rapid social development has also heightened people’s attention to personal health and their demand for a better quality of life, resulting in sufficient research samples and more complete data. Fewer studies in the Western region have examined the relationships between PA, SEM, Self-Rated Health (SRH), and Life Satisfaction (LS) in university students. Existing research primarily focuses on other demographic groups, such as professionals, children, and older people. Additionally, most studies explore the relationships between two or three variables without investigating their interactions in depth (Ma et al., 2002; Tian et al., 2021; Huang et al., 2023). There is a lack of attention to the emotional health of university students in western regions. The global research landscape is similar, with a lack of exploration of the relationship between self-rated health and life satisfaction assessment (Pelletier et al., 2017; Mayes et al., 2022). This study aims to fill this research gap by empirically investigating university students in Western China. It seeks to provide supplementary data and comparisons for related research nationwide. It aims to offer theoretical insights for refining the global health framework and developing targeted health promotion policies.

This study examines two mediating variables, SRH and LS, for the following reasons. SRH is an individual’s expectation and subjective assessment of their health status and is an important indicator of university students’ physical and mental health. It integrates objective health evaluation with subjective health perception and is a common method for international health measurement (Pigott et al., 2024). Enhanced individual self-awareness leads to a deeper understanding of health (Hulsegge et al., 2023). Individuals with higher SRH levels exhibit stable and positive psychological states, possess effective emotional regulation, and have their quality of life assured. LS is a subjective cognitive assessment and emotional experience of one’s life status based on personal standards (Foster and Judy, 2024). Previous research has shown that individuals who actively participate in PA have higher LS (Wypych-Slusarska et al., 2023). They have an optimistic attitude, lighter social anxiety, and improved emotional management by actively enhancing social support (Calleja et al., 2023; Sergio et al., 2024). Moderate LS can affect emotional stability and has a significant role in promoting the generation of positive emotions (Mihaela et al., 2022; Li R. et al., 2024). Self-determination theory (SDT) emphasizes that human behavior is motivated by internal, self-driven factors rather than external environmental influences. Individuals have three basic psychological needs: autonomy, competence, and relatedness. The degree to which these needs are met significantly impacts their motivation and behavior (Ahmadi et al., 2023). Individuals leverage psychological resilience to enhance intrinsic motivation (Zhang et al., 2022), feeling a sense of control over their goals and plans for physical exercise and firmly believing in their ability to achieve these exercise goals (Saad, 2021). While achieving these goals, they establish a connection with their health assessments, consistently expressing vitality and positive emotions, thereby increasing life satisfaction, improving emotional self-regulation, and promoting mental health (Paldam et al., 2019).

**Figure 1:**
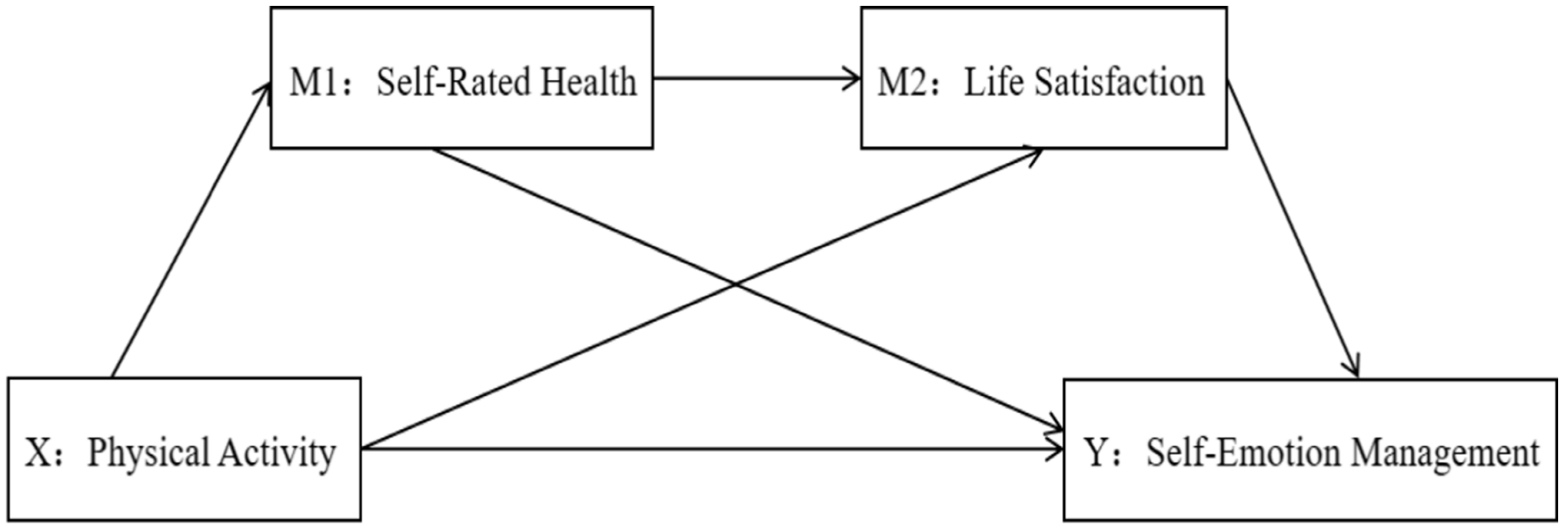
Hypothetical model diagram.

Most existing studies focus on the relationship between SRH or LS and PA and emotions or explore the mediating effects of SRH and LS on PA and other factors (Feiyang et al., 2021; Yu et al., 2024), touching upon their influence on emotional management (Saad, 2021; Daniela et al., 2022). However, these studies often remain at the level of surface correlation analysis and fail to delve into the mechanisms by which SRH and LS function as mediators between PA and emotional management. Based on Self-Determination Theory (SDT), consistent exercise promotes the generation of positive emotions by fulfilling needs for autonomy, competence, and relatedness, thereby enhancing self-regulation of emotions and improving overall mental health among college students. In summary, this study aims to establish a pathway between PA and SEM for college students, grounded in existing literature frameworks on the physiological and psychological mechanisms through which PA influences SEM. Accordingly, Hypothesis *H1: There is a correlation between PA, SRH, LS, and SEM.* The study aims to explore the impact of PA, self-rated health, and life satisfaction on self-emotion management among college students in western China. To further investigate how PA affects emotion regulation abilities, and based on SDT principles, Hypothesis *H2: SRH has a mediating effect between PA and SEM*, *H3: LS has a mediating effect between PA and SEM.* These hypotheses aim to reveal how self-rated health and life satisfaction, as mediating variables, influence PA participation and indirectly affect self-emotion management. College students experience varying intensities of PA and perceive their ability to overcome challenges encountered during different PA intensities, adopting a series of positive mental health behaviors to regulate their emotional changes rationally. Therefore, Hypothesis *H4: SRH and LS sequentially mediate the relationship between PA and SEM.* This hypothesis analyses how self-rated health and life satisfaction interact and serve as key mechanisms through which PA influences self-emotion management. Hypothesis *H5: LS, SRH, SEM, and the intensity of PA are related*, aiming to explore the impact of different PA intensities on relevant mental health indicators (Figure 1).

## 2 Methods

### 2.1 Participants

The primary research method employed in this study is epidemiological investigation. The research encompasses the western regions of China, specifically Sichuan, Chongqing, Guizhou, Yunnan, Shaanxi, Gansu, Qinghai, Ningxia, Xinjiang, Guangxi, and Inner Mongolia. A total of 10,300 valid questionnaires were gathered during this investigation. The target population for this survey is undergraduate students from general higher education institutions in China’s western region; this includes both associate and bachelor’s degree students while excluding postgraduate (master’s and doctoral) candidates. Survey participants were selected using stratified clusters. The cluster sampling methods are based on geographical regions (such as provinces and urban administrative levels) and university types (such as comprehensive universities and local colleges). The specific sampling procedures are outlined as follows:

#### 2.1.1 Determination of sampling sites

To ensure the representativeness of the surveillance subjects, an average of three sampling sites were distributed to each province (region, municipality). Based on the principle of drawing an equal number of samples from different cities, the specific approach is as follows: Prefecture-level cities, governed by provinces and autonomous regions, are designated sampling sites. Among these, the provincial capital is appointed as a “first-class” sampling location; the remaining two cities are selected based on the geographical considerations of the province or autonomous region. A city with an average socio-economic development level is classified as a second-class sampling site. In contrast, a city with lower socio-economic development is classified as a third-class sampling site. In municipalities directly under the central government district, sample collection does not adhere to these principles and primarily relies on random cluster selection. However, it must follow the requirement of three designated sampling sites.

#### 2.1.2 Determination of sampling units

When selecting sampling units, three main aspects were considered: First, the affiliated higher education institutions should be established institutions registered with the Ministry of Education, including vocational colleges; second, units that can meet the sampling requirements (age, number, grade distribution, etc.); third, units with specific questionnaire distributors and a willingness to participate in long-term monitoring. Fourth, the sampling units are affiliated with higher education institutions where university students have completed their return to campus for the autumn semester.

#### 2.1.3 Grouping and sample size

The population is divided into two groups based on sex (This study focuses solely on sex: male or female), then categorized into eight types of samples according to grade, with each type of sample (e.g., male first-year university students) further divided into two categories based on ethnicity (Han, ethnic minorities). To ensure adequate representation of ethnic minorities in the sample, we specifically focused on the distribution characteristics of ethnic minority students in the Western region. We selected institutions with a higher proportion of ethnic minorities and ensured that at least one department within each institution contained a sufficient proportion of ethnic minority students. Furthermore, through the self-reported ethnicity section of the survey, we conducted statistical tests on the essential demographic characteristics of the sample (such as sex, grade, and ethnic proportions). We compared these with the overall distribution of university students in the Western region to validate the diversity and representativeness of the sample.

This study selected 10,300 college students aged 15–28 from the Western region as research subjects (46.1% male and 53.9% female). Questionnaire data collection was conducted online, with researchers and supervisors present. The researchers provided informed consent forms to participants at the beginning of the study. The form clearly outlined the study’s purpose, methods, potential risks, and participants’ rights, ensuring voluntary participation based on full awareness. All participants were informed that the questionnaire would take approximately 10 min. Participants were informed that they could withdraw from the study at any time without any negative consequences. A pre-survey was conducted before the main survey, and the questionnaire design was optimized based on the feedback received. Participants’ responses were collected anonymously, and the sample data were kept confidential to minimize self-report biases. A total of 10,300 samples were collected (Table 1).

**Table 1:** Sample characteristics table.

Variable	*n*	%
Sex		
Male	4,749	46.1
Female	5,551	53.9
Nation		
Han Nationality	8,696	84.4
Ethnic Minorities	1,604	15.6
Age		
Aged 18 and under	3,928	38.1
19	3,850	37.4
20	1,515	14.7
21	596	5.8
22	218	2.1
23	106	1.0
24	38	0.4
Aged 25 and over	49	0.5
Grade		
First-year students	6,860	66.6
Sophomores	2,770	26.9
Juniors	446	4.3
Seniors	224	2.2
Total	10,300	100

### 2.2 Measurement

#### 2.2.1 Sociodemographic information

This encompasses sex, academic year, age, educational institution, and the school’s geographical location [postal code must be provided (Luo et al., 2018)].

#### 2.2.2 Physical activity

The Physical Activity Rating Scale-3 (PARS-3) measured PA participation levels among university students. Developed by Japanese scholar Hiroo Hashimoto (Kimio, 1990) and revised by Chinese scholars such as Qingde Liang into the Chinese version (Liang, 1994), the PARS-3 evaluates PA based on intensity, frequency, and duration of exercise sessions. The rating scale ranges from 1 to 5.

*PA volume = Intensity × (Duration - 1) × Frequency…Formula A.*

The World Health Organization categorizes physical activities into Low-Intensity Physical Activity (LPA), Moderate-Intensity Physical Activity (MPA), and Vigorous-Intensity Physical Activity (VPA) based on intensity (Foster et al., 2018). In PARS-3, each indicator is graded on a scale of three levels, corresponding to LPA, MPA, and VPA. The normative data for Chinese adults on PARS-3 are as follows: LPA scores ≤19, MPA scores range from 20 to 42, and VPA scores ≥43 (Liang, 1994). The results from PARS-3 serve as a metric for PA volume and, to a certain extent, reflect the current state of PA participation among university students within a specific time. The test–retest reliability of PARS-3 is 0.820, and its applicability in the Chinese university student population has been validated in numerous studies (Han et al., 2022; Li et al., 2022; Xu et al., 2023; Mu et al., 2024a).

#### 2.2.3 Self-emotion management

The Emotional Intelligence Scale (EIS) was employed to assess the emotional self-regulation of university students. The EIS, developed by Mayer and Salovey in 1998 and based on the emotional intelligence theory, measures individuals’ abilities to perceive, understand, express, control, manage, and use their own and others’ emotions. The scale consists of 8 items, rated on a scale of 1 to 5. A higher total EIS score indicates a higher level of individual emotional self-regulation ability (Zhang, 2008). Translated and validated by Chinese scholar Caikang Wang in 2002, the EIS has demonstrated good reliability and validity (Cronbach’s *α* = 0.84) and has been widely used and verified by Chinese scholars (Wang, 2002). The applicability of the EIS in the Chinese university student population has been confirmed in multiple studies (Wang et al., 2020, 2022a; Mu et al., 2024a).

#### 2.2.4 Self-rated health

The 36-item Short Form Health Survey (SF-36) was utilized to measure the SRH of university students (Salovey and Mayer, 1990). This study selected the “general health” part of the SF-36 for analysis. The scale uses reverse scoring, consisting of 5 items with rating levels from 1 to 5. The higher the total score obtained by summing the 5 items, the poorer the health status. The reliability and validity of SF-36 in measuring health in university students have been validated. The consistency correlation coefficient is 0.92, and Cronbach’s alpha coefficient is 0.88. The reliability and validity are at a good level for use in practice (Han et al., 2015). The SF-36 has been validated for its applicability in multiple studies of Chinese university student populations (Li B. et al., 2024; Mu et al., 2024b).

#### 2.2.5 Life satisfaction

The Satisfaction with Life Scale (SWLS) was used to measure university students’ LS. Developed by American scholars Diener et al. in the 1980s (Pavot and Diener, 1993). The scale consists of five items rated on a 7-point scale. Higher total scores show greater life satisfaction. The SWLS demonstrates good psychometric properties (Cronbach’s *α* = 0.82) and has been widely used and validated by Chinese scholars. The applicability of the SWLS in the Chinese university student population has been confirmed in multiple studies (Xing et al., 2002; Feiyang et al., 2021).

This study localized the international scales (from English to Chinese) to ensure their applicability to the specific population of university students in Western China. Additionally, several domestic studies validated the language-adjusted scales’ suitability in the Chinese university student population (Wang et al., 2020; Feiyang et al., 2021; Han et al., 2022; Li et al., 2024a).

### 2.3 Statistical analysis

This study employed SPSS 26.0 and Excel software, divided into the following steps:

1. Preliminary data processing of questionnaire data obtained from Questionnaire Star was conducted using Excel software, with retesting or deletion of missing or problematic data.
2. Harman’s single-factor test was used to assess common method bias, preventing potential issues related to common method variance.
3. Descriptive statistical methods, including mean, standard deviation, and percentages, were used to analyze the basic information of the survey respondents, encompassing sex, academic year, ethnicity, school region, PA volume, SRH, LS, and SEM. In the descriptive analysis, differences were evaluated with *η*2 effect sizes categorized as small (0.04), medium (0.25), and large (0.64).
4. Pearson correlation analysis was conducted to examine the relationships between university students’ PA (LPA, MPA, VPA), SRH, LS, and SEM.
5. Mediation effect tests were performed through regression analysis, with the Process plugin used for multiple regression analysis and the Bootstrap method for mediation effect analysis.

## 3 Results

### 3.1 Analysis of normality tests

Scholar Kline indicates that when skewness is between ±3, and kurtosis is between ±10, it suggests that the sample data approximately follows a normal distribution (Kline, 2016). Using SPSS software, normality tests were conducted on the primary research variables PA, SEM, SRH, and LS in this study. The measurement results indicate that all variables meet the criteria, and the sample data can be used for subsequent analysis.

### 3.2 Test for common method bias

To address potential common method bias, Harman’s single-factor test was used to conduct an exploratory factor analysis on all items related to PA, SRH, LS, and SEM abilities, considering the subjective nature of the data collection scales. The results revealed five principal components with eigenvalues >1, with the largest factor explaining 32.57% of the variance, which is below the typical threshold of 40%. Consequently, it was concluded that there is no issue with common method bias in this study.

### 3.3 Descriptive statistics

As shown in Table 2, university students scored an average of 6.008 ± 4.811 in LPA, with significant differences observed in sex and academic year but not in ethnicity. The total score for SEM abilities was 29.360 ± 4.814, with statistically significant differences at the levels of sex and academic year. The overall scores for SRH and LS were 62.942 ± 19.318 and 22.130 ± 6.235, respectively, with minimal differences across sex, ethnicity, and academic year.

**Table 2:** Overview of descriptive analysis results.

Research variables
													LPA	SEM	SRH	LS	MPA	SEM	SRH	LS	VPA	SEM	SRH	LS
Population												
M	6.080	29.360	62.942	22.130	26.060	29.430	62.751	22.140	62.910	29.480	61.411	22.080
SD	4.811	4.814	19.318	6.235	6.815	4.696	19.076	6.193	16.079	4.740	19.254	6.241
Sex												
Male												
M	6.770	29.260	63.050	22.150	26.800	29.370	62.575	22.100	63.690	29.510	61.569	22.020
SD	5.022	4.983	19.220	6.300	6.972	4.634	18.440	6.149	16.669	4.695	19.278	6.221
Female												
M	5.720	29.410	62.885	22.120	24.880	29.510	62.751	22.210	59.890	29.350	60.801	22.310
SD	4.655	4.721	19.372	6.202	6.390	4.794	19.076	6.267	13.175	4.915	19.184	6.324
F	73.589	1.403	0.111	0.042	42.105	0.459	0.046	0.164	12.567	0.259	0.355	0.494
P	<0.001	<0.001	0.739	0.837	<0.001	<0.001	0.830	0.686	<0.001	0.611	0.551	0.482
*η* ^2^	0.011	0.015	<0.001	<0.001	0.019	0.015	<0.001	<0.001	0.009	<0.001	<0.001	<0.001
Nation												
Han nationality												
M	6.030	29.350	62.846	22.140	25.980	29.430	62.587	22.170	63.020	29.450	61.346	22.020
SD	4.825	4.842	19.357	6.274	6.792	4.679	18.618	6.215	15.962	4.655	19.151	6.260
Ethnic Minorities												
M	6.370	29.430	63.493	22.050	26.440	29.420	62.919	22.000	62.380	29.590	61.724	22.40
SD	4.717	4.649	19.093	6.008	6.923	4.783	19.031	6.093	16.662	5.140	19.789	6.157
F	4.237	0.232	0.958	0.174	1.409	0.000	0.097	0.225	0.305	0.149	0.074	0.719
P	0.040	0.630	0.328	0.667	0.235	0.982	0.755	0.635	0.581	0.700	0.786	0.397
*η* ^2^	0.001	<0.001	<0.001	<0.001	0.001	<0.001	<0.001	<0.001	<0.001	<0.001	<0.001	0.001
Grade												
First-year students												
M	6.300	29.350	62.876	22.160	25.675	29.340	62.978	22.070	62.410	29.510	61.509	22.150
SD	4.873	4.807	19.252	6.248	6.717	4.683	18.451	6.162	16.032	4.756	19.432	6.179
Sophomores												
M	5.670	29.350	62.930	22.070	25.680	29.630	61.816	22.350	63.300	29.730	61.469	22.330
SD	4.621	4.753	19.477	6.183	6.909	4.833	19.308	6.250	16.618	4.535	18.803	6.275
Juniors												
M	5.510	29.560	63.728	22.210	27.650	29.790	62.723	23.040	64.810	28.800	60.868	21.540
SD	5.060	5.220	18.614	6.611	7.203	4.508	19.615	6.146	15.308	5.283	20.419	6.555
Seniors												
M	5.509	29.580	63.993	22.020	29.660	29.340	60.366	19.930	64.960	28.790	60.612	20.530
SD	4.571	5.225	20.499	5.989	6.966	3.838	17.153	6.377	15.306	4.178	16.061	6.262
F	9.375	0.242	0.273	0.113	6.297	0.682	0.704	2.737	1.124	1.256	0.069	1.520
P	<0.001	<0.001	0.845	0.952	<0.001	0.043	0.550	0.042	0.338	<0.001	0.997	0.207
*η* ^2^	0.004	0.009	<0.001	<0.001	0.009	0.001	0.001	0.004	0.002	0.013	<0.001	0.003

University students’ scores for MPA averaged 26.060 ± 6.815, with significant sex differences and academic year, but not in ethnicity. The total score for SEM abilities was 29.430 ± 4.696, with significant differences at the levels of sex and academic year. The scores for SRH were 62.751 ± 19.076, and for LS, the total score was 22.140 ± 6.193, with minimal differences across sex, academic year, and ethnicity.

For VPA, university students scored an average of 62.910 ± 16.079, with no differences observed in ethnicity and academic year, but significant sex differences. The total score for SEM abilities was 29.480 ± 4.740, with no significant differences in SRH across sex and ethnicity but differences were noted in the academic year. The total score for LS was 22.080 ± 6.241, with minimal differences across sex, academic year, and ethnicity.

**Figure 2:**
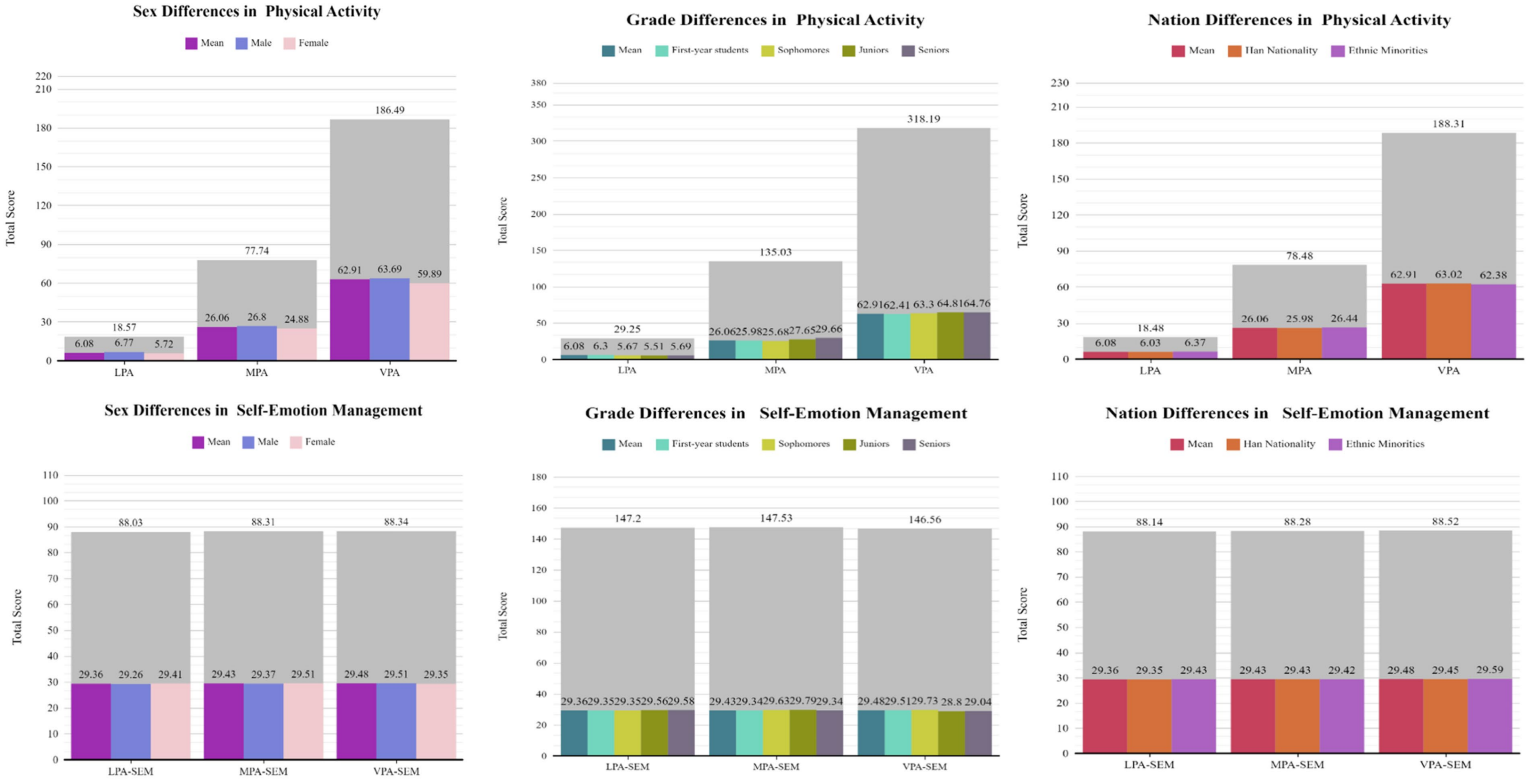
Sex, grade, and nation differences in physical activity intensity and self-emotion management scores.

The study found sex differences among college students in various PA levels (LPA, MPA, VPA) and SEM. Males scored higher than females in all PA levels, and females had higher overall SEM scores than males (Figure 2).

### 3.4 Correlation analysis of the study variables

The Physical Activity Rating Scale (PARS) levels were categorized into LPA, MPA, and VPA and were analyzed about relevant variables (Nie and Heung, 2022). As indicated in Table 3, LPA exhibited significant positive correlations with LS and SEM. At the same time, SRH, LS, and SEM demonstrated significant positive correlations, with SRH and LS showing a notably positive relationship.

**Table 3:** Correlation analysis.

PARS level		RARS total score	SEM	SRH	LS
LPA	RARS total score				
	SEM	0.023*			
	SRH	0.005*	0.309**		
	LS	0.030*	0.572**	0.365**	
MPA	RARS total score				
	SEM	0.006*			
	SRH	−0.019	0.314**		
	LS	0.014	0.553**	0.349**	
VPA	RARS total score				
	SEM	0.018*			
	SRH	−0.012	0.306**		
	LS	−0.016	0.579**	0.373**	

*The correlation is significant at the 0.05 level (two-tailed). **The correlation is significant at the 0.01 level (two-tailed).

VPA showed a significant positive correlation with SEM. SRH, LS, and SEM had significant positive correlations, and SRH and LS showed a significant positive correlation (all *p* < 0.01).

### 3.5 Analysis of regression

**Figure 3:**
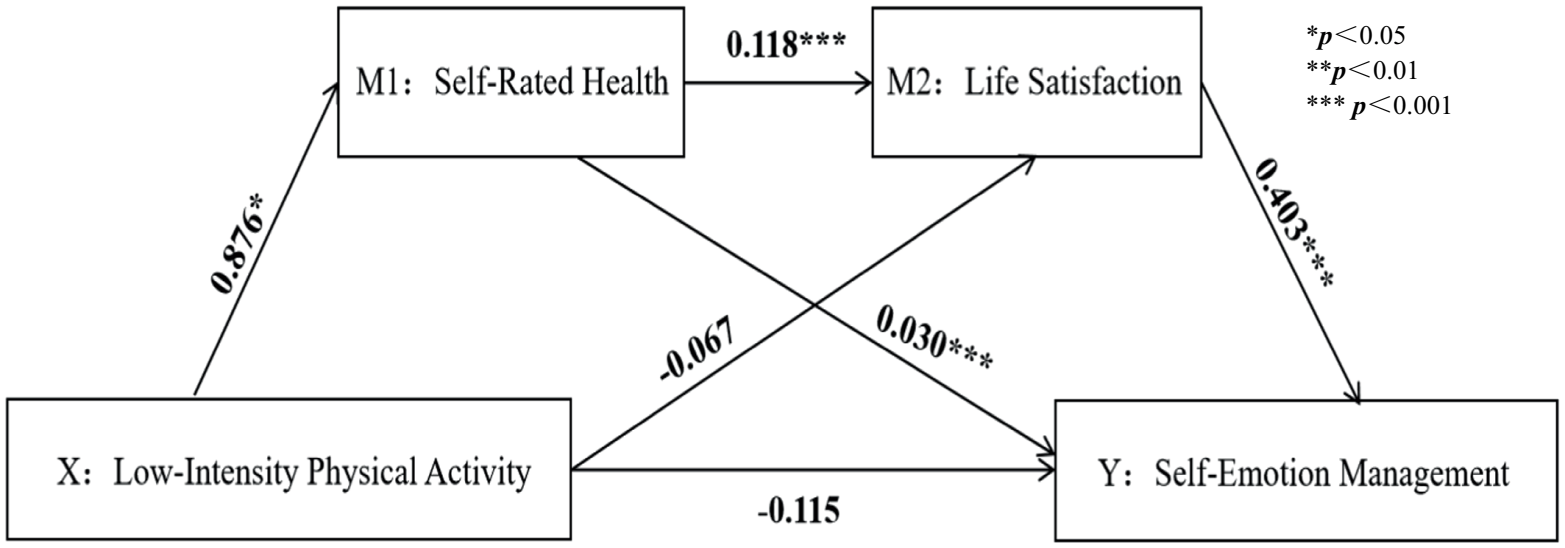
Low-intensity physical activity path analysis diagram.

**Figure 4:**
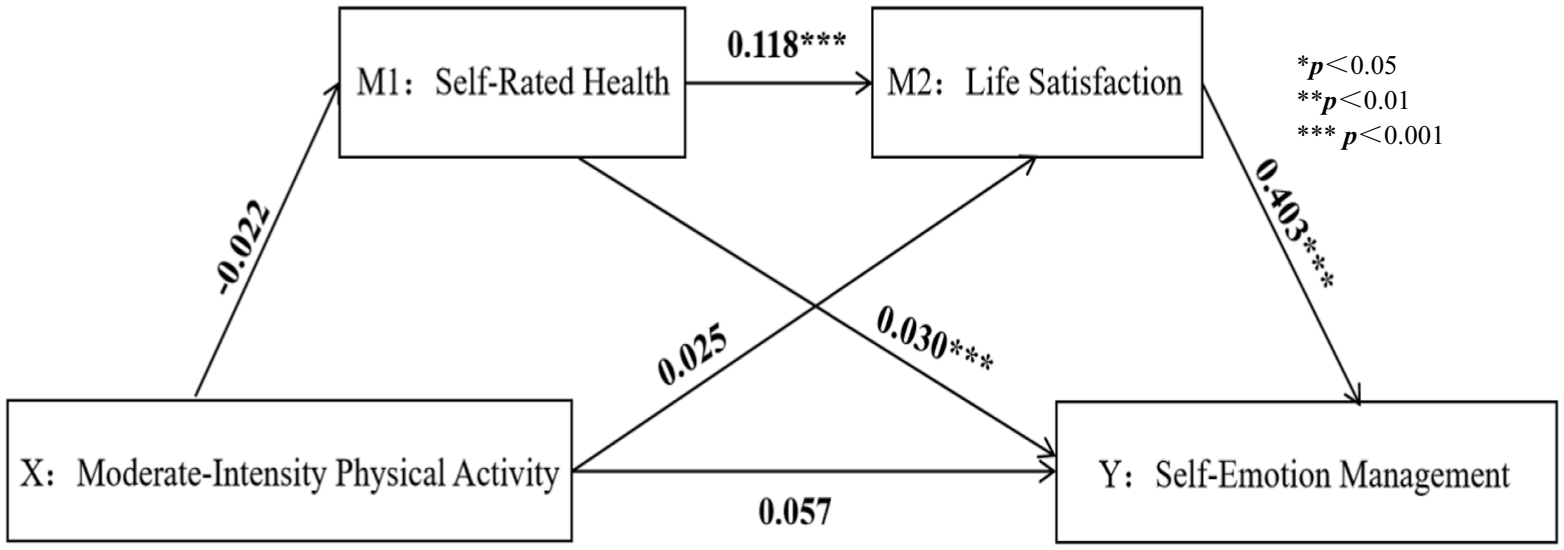
Moderate-intensity physical activity path analysis diagram.

**Figure 5:**
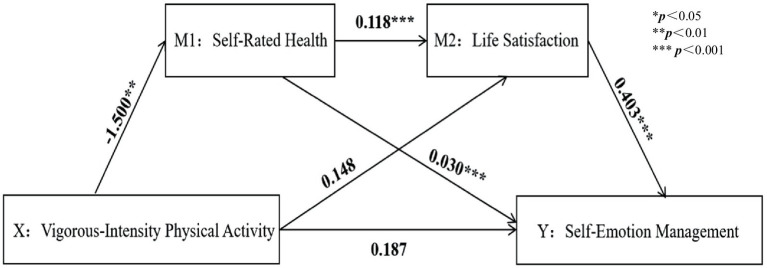
Vigorous-intensity physical activity path analysis diagram.

**Figure 6:**
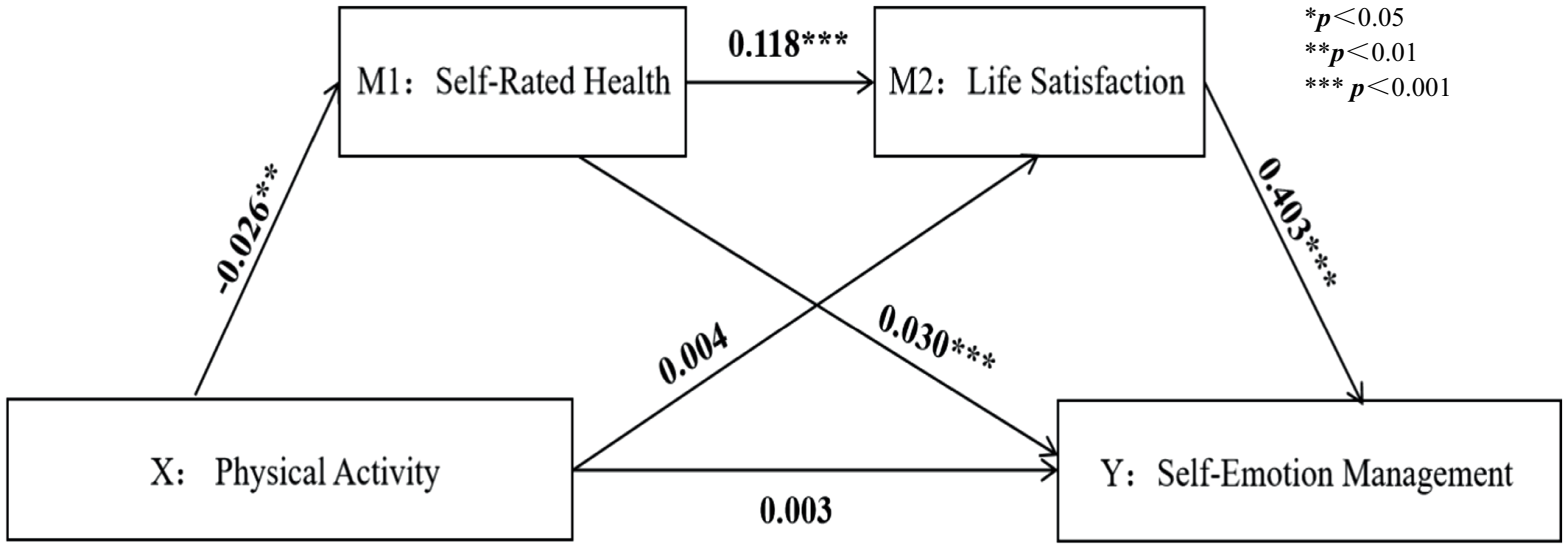
Physical activity path analysis diagram.

A chained mediation model was tested using Model 6 in SPSS 26.0’s PROCESS, controlling for variables such as sex, ethnicity, and grade, as shown in Table 4. The results indicated that LPA positively predicted SRH, while VPA and PA negatively predicted SRH. After incorporating LS into the regression equation, SRH positively predicted LS and SEM. LS significantly positively affected SEM, suggesting that PA might indirectly predict SEM through SRH and LS (all *p* < 0.001) (Figures 3–6).

**Table 4:** Regression analysis of the relationships among variables in the model.

Regression			Fitting indices			Coefficient		
Outcome variables	Predictive variables		*R*	*R^2^*	*F*	*β*	*SE*	*t*
SEM	LPA		0.580	0.336	1221.102***			
						−0.115	0.089	−1.295
		SRH				0.030	0.030	13.807***
		LS				0.403	0.007	60.988***
		Sex				0.077	0.081	0.955
		Nation				0.621	0.106	0.587
		Grade				0.070	0.057	1.239
			0.576	0.329	138.951***			
	MPA					0.057	0.095	0.594
		SRH				0.030	0.002	13.784***
		LS				0.403	0.007	60.989***
		Sex				0.052	0.078	0.666
		Nation				0.066	0.106	0.620
		Grade				0.074	0.057	1.298
			0.587	0.342	118.748***			
	VPA					0.187	0.118	1.589
		SRH				0.030	0.002	13.822***
		LS				0.403	0.007	60.982***
		Sex				0.078	0.080	0.980
		Nation				0.063	0.106	0.597
		Grade				0.667	0.057	1.178
SRH	LPA		0.023	0.005	1.317			
						0.876	0.436	2.010*
		Sex				−0.076	0.400	−0.189
		Nation				0.542	0.522	1.038
		Grade				−0.062	0.279	−0.221
			0.011	0.000	0.308			
	MPA					−0.022	0.469	−0.046
		Sex				0.171	0.385	0.446
		Nation				0.504	0.522	0.966
		Grade				−0.082	0.158	−0.627
			0.028	0.001	1.984			
	VPA					−1.500	0.579	−2.589**
		Sex				−0.095	0.394	−0.242
		Nation				0.535	0.522	1.026
		Grade				−0.034	0.279	−0.121
LS			0.363	0.132	311.642***			
	LPA					−0.061	0.132	−0.462
		SRH				0.118	0.003	39.458***
		Sex				0.402	0.121	0.332
		Nation				−0.100	0.158	−0.639
		Grade				−0.076	0.084	−0.899
			0.363	0.131	311.600***			
	MPA					0.025	0.142	0.178
		SRH				0.118	0.003	39.456***
		Sex				0.026	0.116	0.224
		Nation				−0.099	0.084	−0.879
		Grade				−0.074	0.084	−0.879
			0.363	0.132	311.755***			
	VPA					0.148	0.175	0.841
		SRH				0.118	0.003	39.466***
		Sex				0.049	0.119	0.413
		Nation				−0.101	0.158	−0.642
		Grade				−0.080	0.085	−0.937

*p < 0.05, **p < 0.01, ***p < 0.001.

### 3.6 Analysis of intermediation effects

#### 3.6.1 Direct effects

Based on regression analysis, with VPA as the subject of analysis, the mediation effect testing procedure was followed to examine the coefficient c of VPA on SEM. The coefficient was not significant, indicating the presence of a suppression effect. This coefficient was not significant, indicating a masking effect. The direct effect of VPA is 0.187, with a 95% confidence interval of (−0.044, 0.418). Since the confidence interval includes 0, it indicates a full mediation effect.

#### 3.6.2 Indirect effects

Coefficients a_1_ = −1.500, a_2_ = 0.030; b_1_ = 0.148, b_2_ = 0.403 were evaluated with VPA as the independent variable; among these, a_2_ was not significant, while the other three coefficients were significant, showing a significant indirect effect. The model and the path relationships between variables are depicted in Figure 2.

The Bootstrap method was used to test the mediating effects of SRH and LS, and the results (Table 5) show that the independent mediating effect of SRH (95% CI: −0.080 to −0.010) exerted a negative hindrance, accounting for 34.10% of the total effect. The independent mediating effect of LS (95% CI: −0.080 to −0.200) was insignificant and, therefore, did not play a role. The chained mediation effect between SRH and LS (95% CI: −0.126 to −0.017) is higher than the individual effect, and there is a significant difference between the chained mediation effect and the individual mediation effect of SRH. The chained mediation effect of both also exerts a negative hindering effect, accounting for 54.50% of the total effect. The direct mediating effect of total PA on SRH (95% CI: −0.001–0.000) has a confidence interval that does not include 0, accounting for 26.70% of the total effect; The total chained mediating effect (95% CI: −0.126 to −0.017) also has a confidence interval that does not include 0, accounting for 40.00% of the total effect. In conclusion, the direct mediating effect of total PA on SRH and the total chained mediating effect is significant.

**Table 5:** Analysis of the mediating effects.

PARS Level	Effect	BootSE	95% CI		Proportion of effect
			LLCI	ULCI	
LPA					
Total effect	−0.072	0.109	−0.285	0.141	
Direct effect	−0.115	0.089	−0.288	0.059	
Indirect effect	0.043	0.063	−0.079	0.168	
LPA → SRH → SEM	0.026	0.013	0.002	0.052	36.10%
LPA → LS → SEM	−0.025	0.054	−0.128	0.081	34.20%
LPA → SRH → LS → SEM	0.042	0.020	0.003	0.082	57.80%
MPA					
Total effect	0.065	0.117	−0.164	0.294	
Direct effect	0.057	0.095	−0.130	0.243	
Indirect effect	0.009	0.068	−0.128	0.140	
MPA → SRH → SEM	−0.001	0.014	−0.028	0.026	0.90%
MPA → LS → SEM	0.010	0.056	−0.101	0.118	15.70%
MPA → SRH → LS → SEM	−0.001	0.022	−0.046	0.041	1.60%
VPA					
Total effect	0.131	0.144	−0.152	0.414	
Direct effect	0.187	0.118	−0.044	0.418	
Indirect effect	−0.056	0.083	−0.219	0.109	
VPA → SRH → SEM	−0.045	0.018	−0.080	−0.010	34.10%
VPA → LS → SEM	0.060	0.070	−0.080	0.200	45.50%
VPA → SRH → LS → SEM	−0.071	0.027	−0.126	−0.017	54.50%
PA					
Total effect	0.003	0.002	0.002	0.008	
Direct effect	0.003	0.002	−0.001	0.007	
Indirect effect	−0.001	0.001	−0.003	0.002	
PA → SRH → SEM	−0.001	0.001	−0.002	−0.001	26.70%
PA → LS → SEM	0.002	0.001	−0.001	0.004	56.70%
PA → SRH → LS → SEM	0.001	0.001	0.001	0.002	40.00%

## 4 Discussion

This study reveals the sequential mediating role of SRH and LS in the relationship between PA and SEM in university students, representing a positive exploration of how PA enhances SEM. From a theoretical perspective, while considerable research has been conducted on emotional management, relatively few studies have integrated PA, SRH, and LS to examine their combined effects on SEM. Traditional research often focuses on the impact of only two factors on emotional management. This study’s innovation lies in treating these three as an interconnected and mutually influential system. Exploring their dynamic interaction mechanism from a comprehensive perspective, ultimately influencing SEM. The good physical fitness resulting from physical activity facilitates the improvement of SRH, promoting mental health (Bondarev et al., 2021). SRH includes psychological health assessment and is a known predictor of perceived emotional states (Stefansson et al., 2008; Rissanen et al., 2011). The level of SRH affects LS, which in turn affects emotion management (Putzke et al., 2002). The mechanisms through which these three factors influence emotional management are interconnected. Understanding their intrinsic relationships helps fill gaps in existing research and contributes to developing emotional management theories. Practically, promoting diverse campus activities can encourage students to exercise physically. Conducting psychological counselling courses can help establish a positive health concept. These efforts align with national policies that encourage university students to participate in sports to enhance mental health. Together, they contribute to the physical and mental wellbeing of university students.

### 4.1 Current status of university students’ physical activity

The study reveals that there are significant differences in PA of different intensities across the dimensions of sex, ethnicity, and grade level. In terms of sex, males scored higher than females in PA of various intensities (Figure 2), and this result is consistent with previous research findings (Shu et al., 2024). Based on the role expectation theory, both men and women strive for a sense of social belonging, which gives rise to sex identity (Cardoso et al., 2022), and they are assigned different social expectations (David et al., 2019). Under the long-term influence of sports culture and values, males possess a particular advantage over females in physical fitness and sports ability. Consequently, they often demonstrate a strong interest in intense and physically demanding sports activities. In contrast, influenced by traditional concepts, females tend to prefer low-intensity, social, or flexibility-oriented activities.

From an ethnic perspective, LPA and MPA scores for ethnic minority students are higher than those of Han students. However, under VPA, Han students scored higher than ethnic minority students (Figure 2), which is consistent with previous research findings (Li et al., 2014). Western ethnic minorities are deeply influenced by their geographical environment and traditional culture, having unique traditional sports activities such as wrestling and horse racing. It strengthens physical fitness, provides entertainment, and offers emotional release, contributing to emotion management. With the acceleration of urbanization and the fast pace of life, traditional labor methods of ethnic minorities have been replaced by mechanization and automation, liberating labor but also reducing PA levels (Ye et al., 2019). Traditional sports have been affected by modern sports, and although they have exercise benefits, they have undoubtedly weakened the unique forms of traditional sports activities of ethnic minorities. Modernization has also impacted the cultural identity of ethnic minorities, affecting the participation of the younger generation in traditional activities.

The study found that students’ PA participation levels increased with grade level under MPA and VPA, while under LPA, it showed a declining trend (Figure 2). The former is consistent with previous research results (Claire et al., 2024), while the latter shows a discrepancy. This result may be because as students’ grade levels increase, academic and employment pressures intensify, leading to greater anxiety about the future and a neglect of the importance of LPA for health (Guo et al., 2023). Alternatively, some students may have developed sedentary habits during their college years (e.g., spending long hours in the dorm playing video games or watching TV shows), decreasing interest in PA and reducing participation.

### 4.2 Current status of university students’ self-emotion management

The results indicate a sex difference in college students’ SEM abilities. Female students have higher emotional management levels than male students (Figure 2), consistent with previous research findings (D'Amico and Geraci, 2022). Previous studies indicate that in certain societies, sex stereotypes align with specific role structures (Archer, 1996). In conflict situations, males often display aggressive behaviors, while females tend to be more compliant, reflecting distinct emotional tendencies based on sex (Atienza et al., 2003). Female university students transitioning from adolescence to adulthood, influenced by hormonal changes and neural development (Rong et al., 2024), tend to be more emotionally sensitive than their male counterparts and exhibit a stronger emotional management ability.

The results indicate little ethnic difference in emotional management ability among university students (Figure 2), with Han students scoring slightly higher than ethnic minorities. This may stem from cultural differences between ethnic groups (Li et al., 2023; Xu et al., 2025). Han students, exposed to educational concepts and values early on, align more closely with the school education system (Deng et al., 2019). The Han culture emphasises traits like restraint and control in emotional management, whereas minority cultures may focus more on directly expressing emotions (Li et al., 2023).

The results indicate significant differences in emotional management skills among different grade levels of university students, displaying an inverted “V” trend (Figure 2), which contrasts with previous research findings (Saibani et al., 2013). Previous studies showed that emotion management ability increases overall with grade (Ikpe et al., 2021). The data from this study suggest that this may be because when students first enter college, they are young and lack mature cognitive abilities under the influence of family functions (Peng et al., 2024). They have less independent space and awareness, and their emotion management abilities are weaker. During the middle period, rationality increases with social relationships (Liu et al., 2024). Experience and cognitive levels also improve. These factors lead to varying levels of exercise motivation, active participation in physical activity, and the development of positive peer relationships (Pandya, 2024). Individuals’ emotions are expressed and relieved, and self-emotion management is improved. In the later stages of college, with the widespread adoption of the internet and social media in the information age, external information spreads rapidly and is complex (Jelleli et al., 2024). University students are affected by academic achievement pressure, and they may neglect their emotions, affecting SEM (Hu et al., 2024).

### 4.3 The influence mechanism of physical activity on university students’ self-emotion management

The study results show that different intensities of PA do not directly and positively predict SEM, but they can indirectly predict SEM through the roles of SRH and LS. The correlation between the four supports hypothesis H1 of this study, consistent with previous research results (Huang et al., 2023). Similar studies globally have also found that the relationship between PA and emotional health is complex rather than a simple direct effect. Some studies have validated the mediating role of health-related factors across different age groups and socio-cultural contexts (Morteza et al., 2023; Arimon et al., 2024; Díaz et al., 2024; Küng et al., 2025). In some Asian countries, studies on workplace environments have validated the positive role of participating in PA in alleviating work stress and improving mood (Bischoff et al., 2019; Taylor et al., 2020; Yook, 2020). This further confirms that the findings of this study have a certain universal foundation on a global scale.

This study demonstrates that LPA and VPA significantly impact emotional regulation, consistent with previous research findings (Wang and Hollett, 2022). Engaging in PA can stimulate the release of neurotransmitters such as endorphins and serotonin within the body, which regulate hormone levels and enhance brain function (Riegel et al., 2023), providing immediate benefits (Kim et al., 2020) and positively influencing emotional regulation. A cross-sectional study has pointed out that the nature of exercise may influence PA’s impact on emotions. Aerobic exercise and yoga have shown effectiveness, and moderate to high-intensity exercise has particularly significant improvement effects (Lin and Gao, 2023), consistent with the theory of cognitive benefits (Nakagawa et al., 2020).

In addition, the study found that there may be situations where the influence of certain intensities of PA on emotions is not significant. We need to consider that sex is an important factor affecting the performance of emotional management with PA participation, which is consistent with the results of numerous previous studies (Campos-Uscanga et al., 2022; He and Qiu, 2022). On the one hand, regarding body composition, there are differences in the proportion of fat mass and muscle mass between males and females (Rodríguez-Carmona et al., 2022), which may affect athletic performance. Due to hormonal changes during their special physiological periods, females tend to have greater emotional fluctuations than males, which may influence the emotional benefits of PA at different intensities (Glavin et al., 2021). On the other hand, sex differences are also reflected in exercise motivation and expression patterns (Chu and Koppenhaver, 2025). Males participate in PA more frequently (Gonçalves and Martínez, 2018) and tend to use PA to transfer negative emotions. At the same time, females attach more importance to their appearance (Jones et al., 2021) and are more inclined to use PA for introspection or social interaction to relieve emotions (Meynadier et al., 2025). Research has shown that when females combine mindfulness training with PA, the effect of emotional management is more significant (Amin et al., 2025). When the goals of PA are inconsistent with emotional needs, it may exacerbate the emotional burden and affect emotional management. Notably, grade level may also be an important influencing factor (Rauff and Kumazawa, 2024). Academic pressure and time allocation changes reflect the characteristic patterns of different grade levels (Semenova and Mahlovanyi, 2017; Wang et al., 2020). The results of a study reveal an inverted “U” shape relationship between students’ PA on weekends and their academic performance. Additionally, it shows a sex difference in the optimal exercise duration, with males at 2 h and females at 1 h (Liu G. Q. et al., 2023). The finding of the inverted “U” shape is consistent with another study that investigated the relationship between the total weekly PA duration and mental health (Shimura et al., 2023). The influence of PA on college students’ self-emotion management may be affected by various factors, which in turn impact students’ physical and mental health development.

### 4.4 The chained mediation effect of self-rated health and life satisfaction

This study demonstrates that SRH mediates the relationship between varying intensities of PA and SEM. In contrast, LS does not mediate the relationship between PA intensities and SEM. This study’s hypothesis H3 is partially set up. This is partially different from previous research results (Daniela et al., 2022). Good SRH implies a positive belief in one’s health status, which helps improve emotional states. SRH may have a negative predictive effect on SEM in certain cases. This phenomenon may arise from differences in cultural values and the lack of social support. In some elite sports social environments, there is a highly competitive value system driven by achievement. Even when an individual’s SRH is at a good level, failure to meet societal standards of “success” in career, academics, or social status may cause a significant psychological gap (Ingrid and Jeylan, 2015). This psychological gap can trigger negative emotions such as anxiety and depression, which may interfere with the normal functioning of self-emotion management. In social environments with weaker support networks, individuals may excessively worry about or misinterpret their health status due to a lack of necessary emotional support and feedback (Handique and Jang, 2025). There may even be a tendency to fully attribute health to individual responsibility, thereby negatively affecting SEM (Yao et al., 2025).

LS is a subjective evaluation of life quality based on personal standards, and the emotional improvement brought about by PA for university students is mostly short-term and immediate, while LS is a periodic and long-term stable state (Fernandes et al., 2024). Immediate emotional improvement may neglect the resolution of fundamental issues, such as academic pressure and tense interpersonal relationships. If these fundamental issues are not resolved and reoccur, they may offset the short-term emotional improvement brought about by exercise (Marlies et al., 2015). With social support, university students’ emotions are supported, and long-term goals that align with their values and interests are set (Lee et al., 2024). They gain self-esteem and a sense of achievement through the process. This leads to stable and sustainable emotional improvement. It fosters deep happiness and satisfaction. Furthermore, comprehensive measures are taken to ensure long-term life satisfaction.

The results of this study show that MPA negatively predicts SRH, which seems counterintuitive. In people’s conventional thinking, MPA is often reflected in physical manifestations (e.g., excessive sweating, dehydration, muscle soreness), with short-term visible results. At this point, people tend to believe they have exercised adequately, leading to errors in their health self-assessment. However, in reality, several side effects of MPA are one of the key factors leading to negative health evaluations. MPA requires a high level of physical fitness. Individuals with lower fitness levels may experience adverse physical reactions. In some cases, they may impair cardiovascular function and increase the risk of exercise-related injuries, which can negatively impact overall health. People engage in PA for various motivations, influenced by psychological capital, desiring quick results. Excessive enthusiasm for exercise can also have the opposite effect, leading to exercise addiction, which harms both physical and mental health, thus making MPA negatively predict SRH.

This study demonstrates that SRH and LS serve as chained mediators between LPA, VPA, PA, and SEM. This supports the hypotheses H4 and H5 of this study. This indicates that when college students can control their exercise intensity themselves, they have a clear understanding of their health status and possess the ability to effectively manage and adjust their emotions. Previous studies suggest that as the frequency and intensity of exercise increase, there may be errors in perceiving exercise intensity (Wang et al., 2022b), and individual differences and exercise habits might affect health assessments, blurring the boundaries of emotional states and thereby diverting attention from negative emotions to improve self-emotion management.

The results of this study show that the mediating chain of MPA does not hold and that MPA has a negative prediction for SRH, which seems counterintuitive. In people’s conventional thinking, MPA is often reflected in physical manifestations (e.g., excessive sweating, dehydration, muscle soreness), with short-term visible results. At this point, people tend to believe they have exercised adequately, leading to errors in their health self-assessment. However, in reality, several side effects of MPA are one of the key factors leading to negative health evaluations. MPA requires a high level of physical fitness. Individuals with lower fitness levels may experience adverse physical reactions. In some cases, they may impair cardiovascular function and increase the risk of exercise-related injuries, negatively impacting overall health (Buckley et al., 2019). People engage in PA for various motivations (Kercher et al., 2024), are influenced by psychological capital, and desire quick results. Excessive enthusiasm for exercise can also have the opposite effect, leading to exercise addiction (Sarah et al., 2023), which harms both physical and mental health. According to the World Health Organization guidelines, engaging in 150 min of moderate-intensity exercise or 75 min of vigorous-intensity exercise per week can effectively improve cardiorespiratory endurance, modify body composition, relieve stress and anxiety, and enhance the sense of wellbeing (Gabriel et al., 2022).

Due to evolving health concepts, university students may subjectively perceive their health as declining, yet objective improvements are evident. LS, as an aspect of long-term health outcomes, is closely linked to PA (Dong et al., 2024). The vast and sparsely populated region of Western China provides an ideal environment for PA. Due to the high altitudes in certain areas, moderate PA helps improve the body’s adaptation to low-oxygen environments and enhances cardiovascular and pulmonary function (Seok et al., 2018). People can stroll on the grassland and live in harmony with nature, or participate in some traditional projects unique to Western cultures, such as the “Tuyi Party,” a dance party of the Uyghur ethnic group. This is not only a manifestation of the diversity of forms of PA, but also an interpretation of health in the cultural concepts of some ethnic minorities. Moreover, it is conducive to physical and mental relaxation, stress relief, and the accumulation of positive emotional experiences.

According to the Self-Determination Theory, if college students participate in exercise based on their internal interests and pleasures or to achieve their established goals, they can fully stimulate their internal and external motivations. Most forms of PA are challenging. College students can meet these challenges with strong psychological resilience. After successfully overcoming difficulties, their achievement motivation (Krapp, 2005) can be significantly stimulated. This sense of achievement derived from successful challenges is a powerful driving force to encourage them to continue exercising. During the exercise, college students actively engage in social interactions (Liu and Shi, 2023) and accomplish their goals through teamwork, genuinely experiencing a strong sense of achievement and pride (Chen et al., 2022). This strengthens their social connections, endows them with a profound sense of social belonging, effectively reduces their feelings of loneliness, and significantly enhances their self-efficacy. The enhancement of self-efficacy makes college students full of confidence in their abilities and more inclined to make positive evaluations of their health conditions. Consequently, their sense of happiness in life increases remarkably. When college students have a good physical and mental experience, they will be more proficient in emotion management and improve their self-emotion management ability (Cui and Yang, 2024).

### 4.5 Limitations

The limitations of this study are: (1) SRH and LS are subjective cognitive assessments and are not combined with objective assessments. (2) The study uses subjective recall questionnaires for analysis and does not use precise instrument measurements, which may lead to data errors. (3) This cross-sectional study makes it difficult to infer causal relationships between variables.

The findings provide valuable insights for understanding educational and health promotion strategies in the 21st century. However, future studies need further longitudinal and empirical research to address the existing causal relationship issues.

### 4.6 Practical implications

The study highlights the potential importance of enhancing SEM skills among university students in fast-paced times and explores strategies that may promote mental health through self-rated health and life satisfaction. It is recommended that universities integrate physical activity interventions into their curricula, establish periodic activity check-in tasks, and create incentive mechanisms. Understanding students’ exercise needs, and respecting sex and ethnic differences. Encourage the offering of traditional sports courses with regional characteristics of the West, such as the introduction of Uyghur traditional dance courses in universities in Xinjiang. The abundant natural resources in the West can also be utilized to offer outdoor adventure courses, such as organizing mountain hiking, rock climbing, and other activities in universities in Shanxi, Gansu, and other regions. Universities can collaborate with local outdoor sports clubs and hire professional coaches to ensure the safety and professionalism of the activities.

Universities can collaborate with communities to regularly organize various sports activities (such as community basketball games, and sports meets), set up health awareness boards, and encourage family physical activity interactions to increase students’ PA levels and raise health awareness. Creating a positive social atmosphere for students enhances their sense of belonging, thereby improving LS. Through collaboration between universities, families, and communities, the aim is to increase PA levels while enhancing LS and SRH, promoting the coordinated development of students’ physical and mental health.

## 5 Conclusion

LPA or VPA cannot directly influence university students’ SEM ability. Based on self-determination theory, SRH can independently influence university students’ SEM ability. It can also indirectly influence SEM by incorporating the chained mediation effect of SRH and LS.

## Data Availability

The raw data supporting the conclusions of this article will be made available by the authors, without undue reservation.
